# Mechanisms of doxorubicin-induced drug resistance and drug resistant tumour growth in a murine breast tumour model

**DOI:** 10.1186/s12885-019-5939-z

**Published:** 2019-08-01

**Authors:** Claudia Christowitz, Tanja Davis, Ashwin Isaacs, Gustav van Niekerk, Suzel Hattingh, Anna-Mart Engelbrecht

**Affiliations:** 1 Department of Global Health, Faculty of Medicine and Health Sciences, African Cancer Institute, Stellenbosch University, Cape Town, 8000 South Africa; 2 Department of Physiological Sciences, Faculty of Science, Stellenbosch University, Stellenbosch, 7600 South Africa; 3 Department of Medical Physiology, Faculty of Medicine and Health Sciences, Stellenbosch University, Cape Town, 8000 South Africa

**Keywords:** Breast cancer, Doxorubicin, Drug resistance, Tumour growth, Signalling pathways, ERK

## Abstract

**Background:**

Doxorubicin is currently the most effective chemotherapeutic drug used to treat breast cancer. It has, however, been shown that doxorubicin can induce drug resistance resulting in poor patient prognosis and survival. Studies reported that the interaction between signalling pathways can promote drug resistance through the induction of proliferation, cell cycle progression and prevention of apoptosis. The aim of this study was therefore to determine the effects of doxorubicin on apoptosis signalling, autophagy, the mitogen-activated protein kinase (MAPK)- and phosphoinositide 3-kinase (PI3K)/Akt signalling pathway, cell cycle control, and regulators of the epithelial-mesenchymal transition (EMT) process in murine breast cancer tumours.

**Methods:**

A tumour-bearing mouse model was established by injecting murine E0771 breast cancer cells, suspended in Hank’s Balances Salt Solution and Corning® Matrigel® Basement Membrane Matrix, into female C57BL/6 mice. Fourty-seven mice were randomly divided into three groups, namely tumour control (received Hank’s Balances Salt Solution), low dose doxorubicin (received total of 6 mg/ml doxorubicin) and high dose doxorubicin (received total of 15 mg/ml doxorubicin) groups. A higher tumour growth rate was, however, observed in doxorubicin-treated mice compared to the untreated controls. We therefore compared the expression levels of markers involved in cell death and survival signalling pathways, by means of western blotting and fluorescence-based immunohistochemistry.

**Results:**

Doxorubicin failed to induce cell death, by means of apoptosis or autophagy, and cell cycle arrest, indicating the occurrence of drug resistance and uncontrolled proliferation. Activation of the MAPK/ extracellular-signal-regulated kinase (ERK) pathway contributed to the resistance observed in treated mice, while no significant changes were found with the PI3K/Akt pathway and other MAPK pathways. Significant changes were also observed in cell cycle p21 and DNA replication minichromosome maintenance 2 proteins. No significant changes in EMT markers were observed after doxorubicin treatment.

**Conclusions:**

Our results suggest that doxorubicin-induced drug resistance and tumour growth can occur through the adaptive role of the MAPK/ERK pathway in an effort to protect tumour cells. Previous studies have shown that the efficacy of doxorubicin can be improved by inhibition of the ERK signalling pathway and thereby treatment failure can be overcome.

**Electronic supplementary material:**

The online version of this article (10.1186/s12885-019-5939-z) contains supplementary material, which is available to authorized users.

## Background

Cancer is a major disease burden worldwide and the occurrence of cancer is expected to increase due to the increasing prevalence of lifestyle risk factors and the growth and aging of the population [1]. Based on Global Cancer Incidence, Mortality and Prevalence (GLOBOCAN) estimates, breast cancer was the most frequently diagnosed cancer and the leading cause of cancer deaths among females in 2012 worldwide [1]. Although significant progress has been made regarding treatment options for cancer patients, therapeutic resistance and toxicity of these drugs to normal tissue still remains a major problem. Resistance to chemotherapeutic drugs can cause treatment failure in over 90% of patients with metastatic cancer [2].

Doxorubicin (DXR) is part of the anthracycline family and is currently the most effective chemotherapeutic drug used to treat breast cancer [3, 4]. It has, however, been shown that DXR can induce drug resistance and even tumour growth resulting in poor patient prognosis and survival [5–7]. Although several mechanisms have been investigated, DXR resistance still remains a major unresolved issue in the treatment of cancer patients [8]. Studies reported that the interaction between signalling pathways can promote DXR resistance through the induction of proliferation, cell cycle progression and prevention of apoptosis [5, 9, 10].

The mitogen-activated protein kinase (MAPK)/ extracellular-signal-regulated kinase (ERK) and phosphoinositide 3-kinase (PI3K)/Akt pathways play an essential role in the regulation of proliferation, cell cycle progression, and apoptosis [9, 11]. The MAPK/ERK pathway has been shown to promote DXR resistance through its adaptive role in protecting cancer cells from oxidative stress [12]. Reactive oxygen species (ROS) generation following DXR treatment can also activate other MAPK pathways, including the c-jun N-terminal kinases (JNK) and p38 pathways [12]. The PI3K/Akt pathway can also induce chemo-resistance and promote tumorigenesis by phosphorylating various downstream substrates involved in cell survival, cell cycle, cell metabolism, gene transcription and protein synthesis [13].

In addition to the mechanism of DXR to induce apoptosis, DXR-mediated DNA damage can also induce cell cycle arrest [14]. This can occur through the activation of the tumour suppressor p53, which regulates the transcription of various genes, including p21 and p16, that are involved in cell cycle control, DNA repair and apoptosis [14, 15]. Defects in these regulators can lead to the failure of DXR to induce cell cycle arrest and thereby promote DXR resistance [16, 17].

Besides the effects of proliferation markers and cell cycle regulators on drug resistance, the epithelial-mesenchymal transition (EMT) process has also been shown to play a role in drug resistance by inhibiting apoptosis and preventing senescence [18]. Biomarkers of the EMT process include the cell surface protein, E-cadherin, cytoskeletal proteins, such as alpha smooth muscle actin (α-SMA) and Vimentin, and the transcription factor, Snail [19]. This process can be activated by the MAPK/ERK and PI3K/Akt signalling pathways, which further emphasises the interaction between various pathways and drug resistance [20].

Another process that can be involved in drug resistance is autophagy [21, 22]. Autophagy, also known as macroautophagy, plays a role in maintaining intracellular homeostasis and survival by degrading damaged proteins and organelles and recycling their components to regenerate metabolic precursors [21]. It has been shown that autophagy can drive cancer cells to acquired chemo-resistance by preventing cellular damage and protecting cancer cells against apoptosis [10, 23]. However, when autophagy is induced by excessive cellular stress it can lead to the upregulation of apoptosis and autophagy dependent cell death (ADCD) [10, 23].

Elucidating the mechanism by which these pathways promote drug resistance can improve the efficacy of chemotherapeutic drugs and overcome treatment failure. The aim of this study was therefore to determine the effects of doxorubicin on apoptosis signalling, autophagy, the MAPK- and PI3K/Akt signalling pathway, cell cycle control and regulators of the EMT process in murine breast cancer tumours. We compared the expression levels of markers involved in cell death and survival signalling pathways, by means of western blotting and fluorescence-based immunohistochemistry, in the breast tumours of mice treated with low and high doses of doxorubicin. Our results suggest that DXR-induced drug resistance and tumour growth can occur through the adaptive role of the MAPK/ERK pathway in an effort to protect tumour cells. Previous studies have shown that the efficacy of doxorubicin can be improved by inhibition of the ERK signalling pathway and thereby treatment failure can be overcome [6, 24–26].

## Methods

### Animal model

Ethical clearance was obtained from the Stellenbosch University Ethical Committee (no. SU-ACUM13–00027). Six-week old female wildtype C57BL/6 mice were obtained from the Tygerberg animal facility. The mice were kept under temperature controlled conditions, underwent a reverse dark-light cycle and were provided standard mouse pellets and tap-water ad libitum in individually ventilated cages (IVC) with autoclaved bedding at the Stellenbosch University animal facility.

Murine E0771 breast cancer cells, syngeneic to C57BL/6 mice, suspended in Hank’s Balances Salt Solution (HBSS, Sigma-Aldrich, MO, USA) were added to Corning® Matrigel® Basement Membrane Matrix (9.2 mg/ml protein, BD Bio-sciences, CA, USA) in a 1:1 ratio. When the mice reached an age of 10 weeks (21.9 g ± 0.25 g), 100 μl was injected subcutaneously at the fourth mammary fat pad of the mice to initiate tumour growth. Tumour growth was monitored every second day and length and width measurements were taken to calculate tumour volume (mm^3^) with the following equation: (length x width^2^)/2.

DXR treatment were initiated once the tumours were palpable (between day 6 and day 9). Forty-seven mice were randomly divided into three groups, namely TC (*n* = 15), LD-DXR (*n* = 16) and HD-DXR (n = 16) groups. The sample size were based on previously unpublished studies. Three doses of DXR were administered three days apart by means of intraperitoneal injection. The TC group received HBSS, the LD-DXR group received 2 mg/kg DXR (total of 6 mg/kg) and the HD-DXR group received 5 mg/kg DXR (total of 15 mg/kg). The dosages were based on previously unpublished studies.

The endpoint of the study was reached when the tumours reached a volume of 400 mm^3^. Mice were anaesthetised with Isofluorane (Isofor, Safeline Pharmaceuticals, RSA) before exsanguination. The tumours were then excised and cut into two parts to be used for western blotting and immunohistochemistry. The samples used for western blotting were snap-frozen in liquid nitrogen and stored at − 80 °C. The samples used for immunohistochemistry were mounted with tissue freezing medium (Leica Biosystems, Germany, UK) and frozen in ice-cold isopentane and stored at − 80 °C.

### Western blots

Standard Radioimmunoprecipitation assay (RIPA) buffer was used to harvest protein lysates from tumour tissues while a Bradford assay was performed to determine protein concentration. Protein samples (50 μg), prepared with Laemmli’s sample buffer, were loaded onto 4–20% Criterion™ TGX Stain-Free™ Precast Gels (BioRad, CA, USA). Proteins were separated at 100 V for 10 min and 120 V for 60 min in Tris/Glycine/SDS running buffer (BioRad, CA, USA). Proteins were transferred onto Polyvinylidene difluoride (PVDF) membranes (Trans-Blot® Turbo RTA Midi PVDF transfer kit, BioRad, CA, USA) with the Trans-Blot® Turbo Transfer System (BioRad, CA, USA) with the conditions of 25 V, 1.0A, 30 min. The Stain-Free™ properties of the membranes were utilized on the Chemidoc™ MP System (BioRad, CA, USA) to determine the total protein intensities of each membrane. Membranes were blocked in 5% milk for 1 h and incubated in primary antibody, prepared in tris-buffered saline with tween 20 (TBS-T), at 4 °C overnight. On the following day membranes were incubated in secondary antibody, prepared in TBS-T, for 1 h at RT. Antibody details are listed in the Additional file 1. After incubation, membranes were developed on the ChemiDoc™ MP System with Clarity™ Electrochemiluminescence (ECL) Substrate (BioRad, CA, USA).

### Fluorescence-based immunohistochemistry

Tumour tissues were sectioned into 8 μm sections with a Leica CM110 Cryostat (Leica Biosystems, Germany, UK). The sections were fixed in 100% ice-cold methanol for 5 min and blocked in 2.5% goat serum, prepared in phosphate-buffered saline (PBS), for 1 h. Sections were covered with p-ERK primary antibody, prepared in PBS, and incubated at 4 °C overnight or with p21 primary antibody, prepared in 2.5% goat serum, and incubated for 1 h at RT. The sections for the secondary antibody control were covered in PBS. On the following day sections were covered in fluorescein isothiocyanate (FITC) secondary antibody, prepared in PBS or 2.5% goat serum, and incubated for 1 h at RT. Antibody details are listed in the Additional file 1. The sections were stained with 10 μg/ml Hoechst 33342 (Sigma-Aldrich, MO, USA), prepared in PBS (1:200), for 15 min at RT. Coverslips were mounted onto the slides with Dako Fluorescence Mounting Medium (Agilent Technologies, CA, USA). Slides were protected from light and stored at − 20 °C until imaging. Five images per section were obtained with the Nikon NIS-Element BR v4.10.00 imaging software on the Nikon Eclipse E400 microscope using the 40x objective. The 405 nm and 488 nm excitation lasers for Hoechst and FITC, detected in the emission ranges of 450–490 nm and 520–540 nm, respectively, were used.

### Statistical analyses

An analysis of covariance (ANCOVA) (*p* < 0.05) was performed to compare the tumour volume between the TC (*n* = 15), LD-DXR (*n* = 16) and HD-DXR (*n* = 16) groups. The western blot experiments were conducted with technical repeats of *n* = 2 and biological repeats of *n* = 8. Bio-Rad Image Lab™ software v5.1 was used for normalization of the protein specific intensities against total protein intensities. Results were analysed in GraphPad Prism v7.0 by performing a one-way analysis of variance (ANOVA) with Bonferonni post hoc test (*p* < 0.05). The mean values ± standard error of the mean was reported. The fluorescent-based immunohistochemistry experiments were conducted with biological repeats of n = 8. Five images per section were analysed in Image J software v1.52a. The following equation was used to calculate the corrected total cryosection fluorescence: mean of integrated density – (mean of area of selected cell x mean fluorescence of background readings).

## Results

### Higher tumour growth rate was observed after DXR treatment

**Fig. 1:**
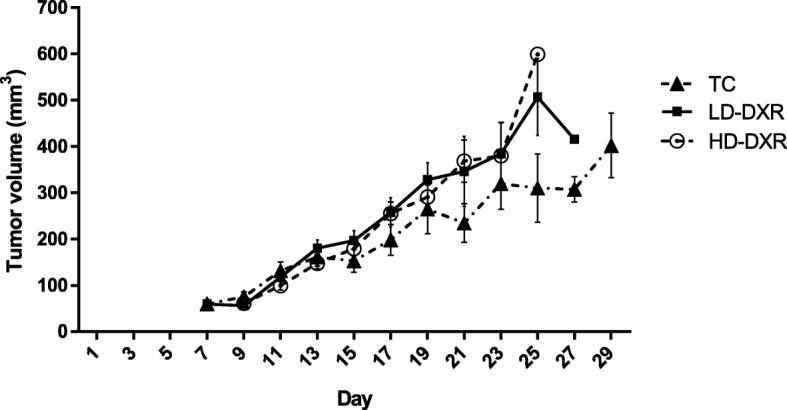
Effects of DXR treatment on the average tumour volume (mm^3^). DXR treatment were initiated between day 6 and day 9. Three doses of DXR were administered three days apart. Error bars indicate the standard error of the mean (*n* = 15 (TC group), *n* = 16 (LD-DXR and HD-DXR groups)). The slope of the regression lines from the LD-DXR and HD-DXR groups were significantly different compared to the TC group. [31]

A tumour-bearing mouse model was established by injecting murine breast cancer cells, suspended in Corning® Matrigel®, into female mice. When the tumours were palpable, the mice either received a vehicle treatment, a low dose of DXR or a high dose of DXR treatment. The tumours grew rapidly over the study period and showed resistance to both DXR doses. Analyses revealed that both low dose (LD)-DXR and high dose (HD)-DXR groups has a significantly increased tumour volume when compared to the tumour control (TC) group (Fig. 1). No significant differences in tumour volume were observed between the LD-DXR and HD-DXR groups (Fig. 1).

### Inability of DXR to induce apoptosis or autophagy, indicating the occurrence of DXR resistance

**Fig. 2:**
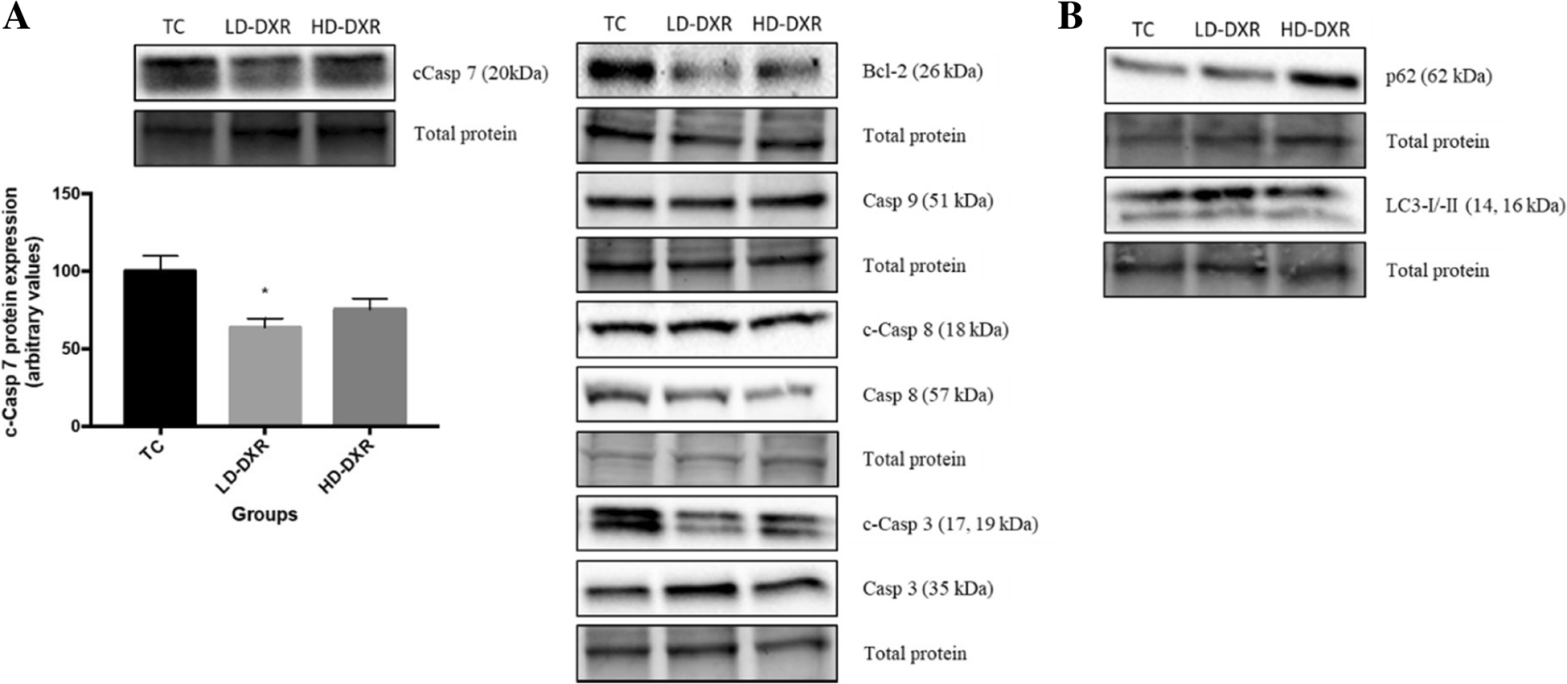
**a** Protein expression of c-Casp 7 between the TC, LD-DXR and HD-DXR groups (*n* = 8). * - significantly different compared to TC group (*p* < 0.05). Representative images of Bcl-2, Casp 9, c-Casp 8, Casp 8, c-Casp 3 and Casp 3 protein expression between the TC, LD-DXR and HD-DXR groups (*n* = 8). **b** Representative images of p62 and LC3-I/−II protein expression between the TC, LD-DXR and HD-DXR groups (*n* = 8)

To determine whether apoptosis was induced after DXR treatment, western blot experiments were performed to compare the protein expression levels of different apoptotic markers, including B-cell lymphoma 2 (Bcl-2), caspase (Casp)-9, Casp-8, Casp-3 and Casp-7 between the different groups. c-Casp 7 protein expression decreased significantly in tumour-bearing mice treated with a low dose of DXR, whereas a non-significant decrease was observed in mice treated with a high dose of DXR (Fig. 2a). No significant differences in the other apoptotic markers were observed between the different groups. Bcl-2 protein expression showed a slight decrease in the LD-DXR and HD-DXR groups compared to the TC group (Fig. 2a). Casp-8 showed a greater decrease in protein expression levels after DXR treatment, than Casp-9 (Fig. 2a). The ratio between cleaved Casp-3 and Casp-3 showed a decrease in both treatment groups compared to the control, with the LD-DXR group showing the lowest protein expression (results not shown). To determine whether autophagy was induced after DXR treatment, western blot experiments were performed to compare the protein expression levels of markers, p62 and microtubule-associated protein light chain 3 (LC3)-I/−II, between the TC, LD-DXR and HD-DXR groups. No significant differences were again observed between the different groups (Fig. 2b).

### The MAPK/ERK pathway had a greater effect on DXR resistance than the PI3K/Akt pathway and other MAPK pathways

**Fig. 3:**
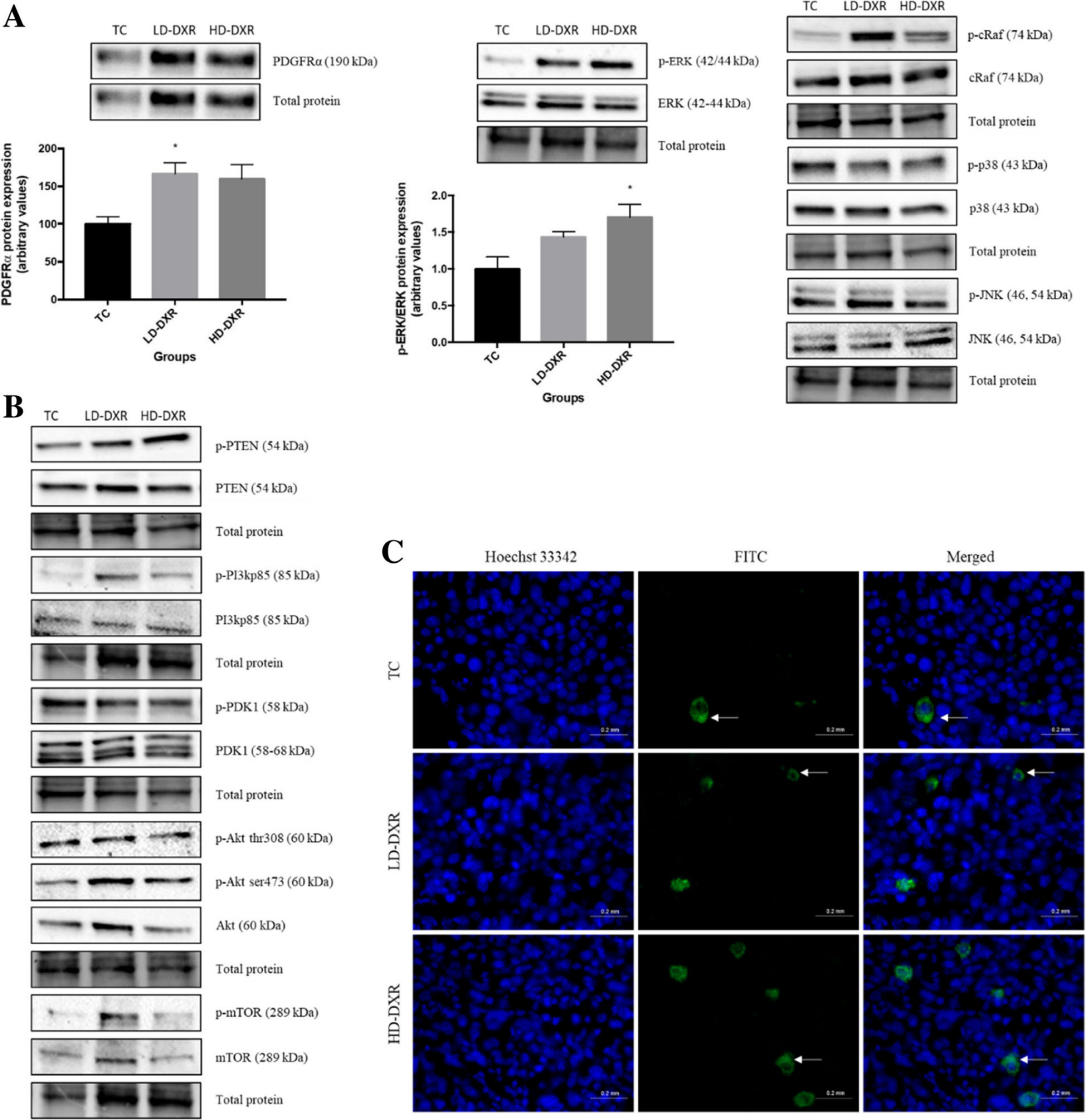
**a** Protein expression of PDGFRα and p-ERK/ERK between the TC, LD-DXR and HD-DXR groups (*n* = 8). * - significantly different compared to TC group (*p* < 0.05). Representative images of p-cRaf, cRaf, p-p38, p38, p-JNK and JNK protein expressions between the TC, LD-DXR and HD-DXR groups (*n* = 8). **b** Representative images of p-PTEN, PTEN, p-PI3Kp85, PI3Kp85, p-PDK1, PDK1, p-Akt thr308, p-Akt ser473, Akt, p-mTOR and mTOR protein expressions between the TC, LD-DXR and HD-DXR groups (*n* = 8). **c** Representative images of p-ERK-FITC signal in tumour tissues following DXR treatment (*n* = 8). Hoechst 33342 – nuclei; FITC – p-ERK; solid arrows – localised areas of intense signal in cytoplasm; scale = 20 μm, 40x objective

To determine the effects of the MAPK/ERK signalling pathway on DXR resistance, we performed western blot experiments to compare the protein expression levels of markers, platelet-derived growth factor receptor alpha (PDGFRα), c-Raf and ERK, between the different groups. We also assessed other MAPK pathways, including the p38 and JNK pathways. PDGFRα protein expression increased significantly in tumour-bearing mice treated with a low dose of DXR, whereas a non-significant increase was observed in mice treated with a high dose of DXR (Fig. 3a). The ratio between phosphorylated ERK (p-ERK) and total ERK showed a gradual increase in protein expression levels as the dosage of DXR increased, with the HD-DXR group showing a significant increase compared to the TC group (Fig. 3a). Fluorescence-based immunohistochemistry was performed to support the western blot results of p-ERK. However, no significant differences in p-ERK signal was observed between the different groups (Fig. 3c). No significant differences were observed in the protein expression levels of c-Raf, p38 and JNK between the different groups (Fig. 3a). To determine the effects of the PI3K/Akt/mammalian target of rapamycin (mTOR) pathway on DXR resistance, western blot experiments were performed to compare the protein expression level of markers, phosphatase and tensin homolog (PTEN), PI3Kp85, phosphoinositide-dependent kinase-1 (PDK1), Akt and mTOR, between the different groups. There were, however, no significant differences in protein expression observed between the different groups (Fig. 3b).

### Changes observed in cell cycle regulators after DXR treatment

**Fig. 4:**
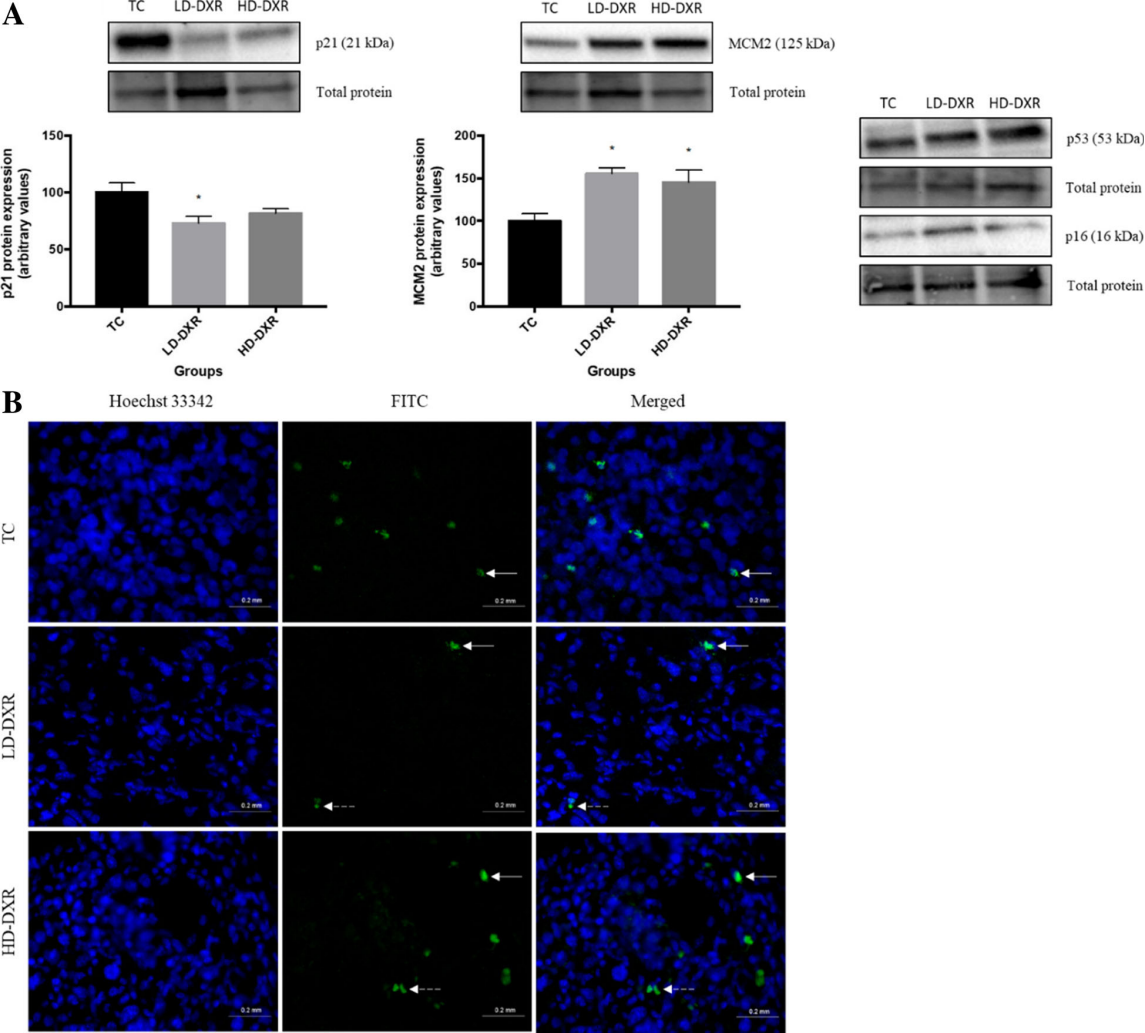
**a** Protein expression of p21 and MCM2 between the TC, LD-DXR and HD-DXR groups (*n* = 8). * - significantly different compared to TC group (*p* < 0.05). Representative images of p16 and p53 protein expressions between the TC, LD-DXR and HD-DXR groups (*n* = 8). **b** Representative images of p21-FITC signal and localisation in tumour tissues following DXR treatment (*n* = 8). Hoechst 33342 – nuclei; FITC – p21; solid arrows – localised areas of intense signal in nuclei; dashed arrows- localised areas of intense signal in cytoplasm; scale = 20 μm, 40x objective

Western blot experiments were performed to compare the protein expression levels of different markers involved in the cell cycle and DNA replication between the TC, LD-DXR and HD-DXR groups. These markers included the tumour suppressor p53, cyclin-dependent kinase (CDK) inhibitors, p21 and p16, and the proliferation marker, minichromosome maintenance 2 (MCM2). A significant reduction in p21 expression was observed in the LD-DXR group compared to the TC group, while a non-significant reduction in p21 expression was observed in the HD-DXR group (Fig. 4a). Fluorescence-based immunohistochemistry was performed to support the western blot results of p21 qualitatively (Fig. 4b). Cytoplasmic signal of inactive p21 was also observed in the LD-DXR and HD-DXR groups (Fig. 4b). MCM2 protein expression increased significantly in both treatment groups compared to the control group (Fig. 4a). No significant differences were observed in the protein expression levels of p53 and p16 between the different groups (Fig. 4a).

### EMT did not occur during DXR-induced drug resistance and tumour growth

**Fig. 5:**
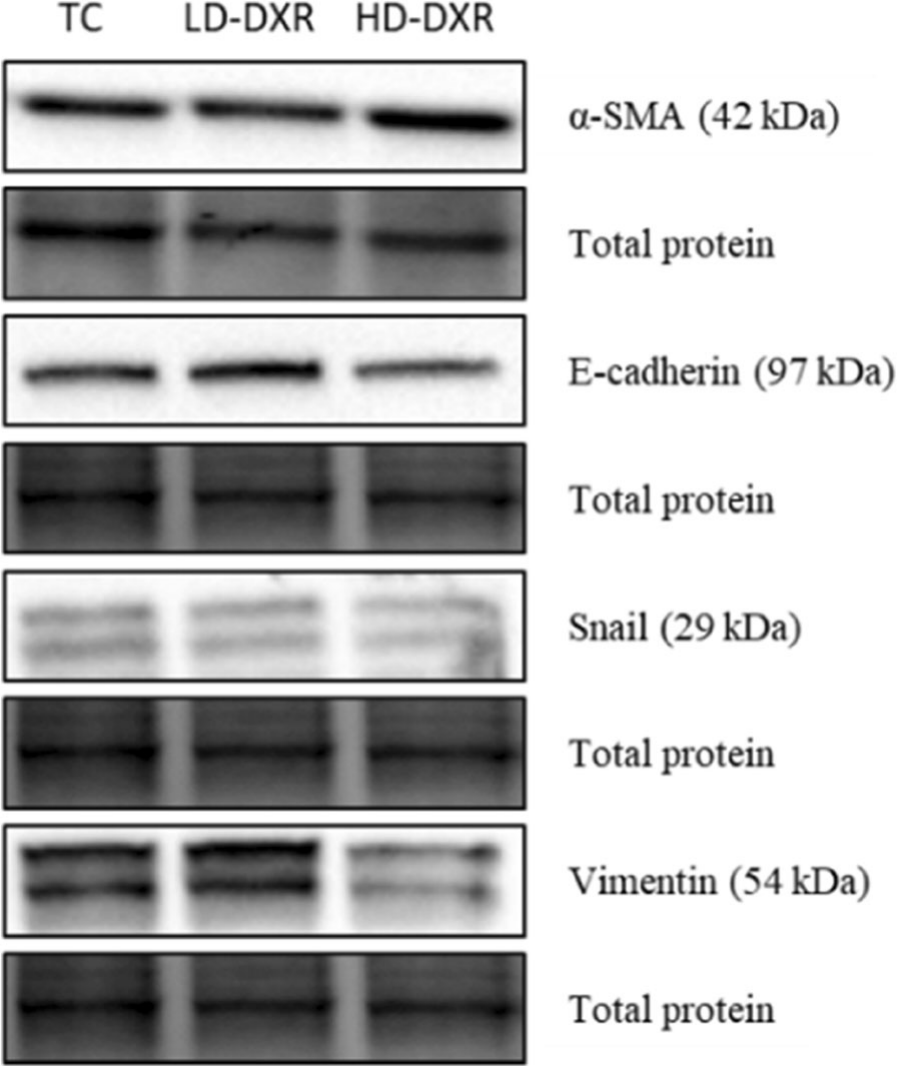
Representative images of α-SMA, E-cadherin, Snail and Vimentin protein expressions between the TC, LD-DXR and HD-DXR groups (*n* = 8)

Western blot experiments were performed to compare the protein expression levels of the EMT markers, α-SMA, E-cadherin, Snail and Vimentin, between the TC, LD-DXR and HD-DXR groups. No significant differences were however observed between the different groups (Fig. 5).

## Discussion

DXR can induce drug resistance and even tumour growth resulting in poor patient prognosis and survival [5–7]. Although several mechanisms have been investigated, DXR resistance still remains a major unresolved issue in the treatment of cancer patients [8]. Studies have shown that the interaction between signalling pathways can promote drug resistance through the induction of proliferation, cell cycle progression and prevention of apoptosis [5, 9, 10].

The aim of this study was therefore to determine the effects of doxorubicin on apoptosis signalling, autophagy, the MAPK- and PI3K/Akt signalling pathway, cell cycle control, and regulators of the EMT process in murine breast cancer tumours. We compared the expression levels of markers involved in cell death and survival signalling pathways, by means of western blotting and fluorescence-based immunohistochemistry, in the breast tumours of mice treated with low and high doses of doxorubicin.

To determine whether cell death was induced after DXR treatment we assessed the expression levels of different markers involved in apoptosis and autophagy. A significant decrease in c-Casp 7 protein expression was observed in tumour-bearing mice treated with a low dose of DXR, whereas a non-significant decrease was observed in mice treated with a high dose of DXR (Fig. 2a). An increase in p62 protein expression was observed in the HD-DXR group, however, not significant (Fig. 2b). No significant changes in LC3 protein expression was observed after DXR treatment (Fig. 2b). We therefore showed that DXR failed to induce cell death through apoptosis or ADCD, indicating the occurrence of drug resistance. It is well known that chemo-resistance is associated with the inability of chemotherapeutic drugs to induce cell death [10, 23].

Interactions between MAPK/ERK and PI3K/Akt signalling pathways play an essential role in the regulation of proliferation, cell cycle progression and apoptosis [9, 11]. The mechanisms by which the MAPK/ERK and PI3K/Akt signalling pathways regulate DXR-induced cell death and drug resistance are, however, controversial [5]. Some studies have shown that sustained ERK activation contributes to DXR-induced cell death and can be negatively regulated by the PI3K/Akt pathway [5, 6].

In contrast, other studies have reported that ERK activation can protect cells from DXR-induced cell death [6, 12]. The MAPK/ERK pathway has been shown to promote DXR resistance through its adaptive role in protecting cells from oxidative stress [12]. ROS generation following DXR treatment can activate PDGFRα, thereby increasing the activation of the MAPK/ERK pathway as well as other MAPK pathways, including the JNK and p38 pathways [12].

A study done by Jin et al. showed that the PI3K/Akt pathway had a greater effect on chemo-resistance than the MAPK pathway in breast cancer cells [27]. We assessed the expression levels of markers associated with the MAPK pathway, such as PDGFRα, cRaf, ERK, p38 and JNK, and markers associated with the PI3K/Akt pathway, such as PTEN, PI3Kp85, PDK1, Akt, and mTOR, to determine which pathway was responsible for DXR-induced drug resistance and tumour growth in the tumour-bearing mouse model. We observed increased protein expression for both PDGFRα and p-ERK and no significant changes in markers of the PI3K/Akt pathway and other MAPK pathways, indicating that the MAPK/ERK pathway had a greater effect on DXR resistance (Fig. 3).

In addition to the mechanism of DXR to induce apoptosis, DXR-mediated DNA damage can also induce cell cycle arrest [14]. This can occur through the activation of the tumour suppressor p53, which regulates the transcription of various genes involved in cell cycle control, DNA repair and apoptosis [14]. CDK inhibitors, such as p21 and p16, are major targets of p53 and is known to function as mediators of DXR-induced cell cycle arrest [14–17].

We therefore assessed the expression level of different cell cycle regulators, such as p53, p21 and p16, to determine whether DXR was able to induce cell cycle arrest. A significant reduction in p21 expression was observed in the LD-DXR group compared to the TC group, while a non-significant reduction in p21 expression was observed in the HD-DXR group (Fig. 4a). p21 is an inhibitor of CDKs and activation of this protein can induce cell cycle arrest. Downregulation of this protein may therefore indicate that DXR was unable to induce cell cycle arrest. Hwang et al. showed that sustained activation of ERK2 can downregulate p21, resulting in cell cycle progression [28]. We therefore suggest that the significant increase observed in ERK expression after DXR treatment (Fig. 3a), contributed to the reduction in p21 expression (Fig. 4a).

MCM2 is a marker of proliferation and plays an essential role in DNA replication [29]. The increase in MCM2 expression observed after DXR treatment indicated that cell cycle arrest did not occur and that the cancer cells were actively proliferating (Fig. 3a). This increase in proliferation supports the increased tumour growth observed in both low and high doses of DXR.

It has been suggested that EMT can also promote chemo-resistance in breast cancer tumours [18, 30]. Since we did not observe any significant changes in the expression levels of the cell surface protein, E-cadherin, the cytoskeletal proteins, such as α-SMA and Vimentin, and the transcription factor, Snail, we propose that EMT did not contribute to the DXR-resistance and tumour growth observed in our study (Fig. 5).

## Conclusions

In conclusion, our results suggest that DXR-induced drug resistance and tumour growth can occur through the adaptive role of the MAPK/ERK pathway in an effort to protect tumour cells. Previous studies have shown that the efficacy of doxorubicin can be improved by inhibition of the ERK signalling pathway and thereby treatment failure can be overcome [6, 24–26].

## Additional file

Additional file 1: **Table S1.** Primary and secondary antibody details. **Table S2.** Characteristics of mice. (DOCX 28 kb)

## Data Availability

The datasets used and analysed during the current study are available from the corresponding author on reasonable request.

## Abbreviations

ADCD: Autophagy dependent cell death
ANCOVA: Analysis of covariance
ANOVA: Analysis of variance
Bcl-2: B-cell lymphoma 2
Casp: Caspase
CDK: Cyclin-dependent kinase
DXR: Doxorubicin
ECL: Electrochemiluminescence
EMT: Epithelial-mesenchymal transition
ERK: Extracellular-signal-regulated kinase
FITC: Fluorescein isothiocyanate
GLOBOCAN: Global cancer incidence, mortality and prevalence
HBSS: Hank’s balanced salt solution
HD-DXR: High dose DXR
JNK: c-jun N-terminal kinases
LC3: microtubule-associated protein light chain 3
LD-DXR: Low dose DXR
MAPK: Mitogen-activated protein kinase
MCM2: Minichromosome maintenance 2
mTOR: mammalian target of rapamycin
PBS: Phosphate-buffered saline
PDGFRα: Platelet-derived growth factor receptor alpha
PDK1: Phosphoinositide-dependent kinase-1
PI3K: Phosphoinositide 3-kinase
PTEN: Phosphatase and tensin homolog
PVDF: Polyvinylidene difluoride
RIPA: Radioimmunoprecipitation assay
ROS: Reactive oxygen species
TBS-T: Tris-buffered saline with tween 20
TC: Tumour control
α-SMA: Alpha smooth muscle actin
