# Supplementary material for: Mechanisms of doxorubicin-induced drug resistance and drug resistant tumour growth in a murine breast tumour model
Source: BMC Cancer. 2019 Aug 1;19:757. doi: 10.1186/s12885-019-5939-z (PMC6670209; doi:10.1186/s12885-019-5939-z)
Supplement: Supplementary file 1 — Table S1. Primary and secondary antibody details. Table S2. Characteristics of mice. (DOCX 28 kb) [file 12885_2019_5939_MOESM1_ESM.docx]

**Additional file 1**

**Table S1.** Primary and secondary antibody details

| **Primary Antibody**  **(1° AB)** | **Size** | **Company** | **Catalogue Number** | **Specie** | **1° AB Dilution** | **Secondary Antibody (2° AB)** | **2° AB Dilution** |
| --- | --- | --- | --- | --- | --- | --- | --- |
| PTEN | 54 | Cell Signaling Technology | #9559 | Rabbit | 1: 1000 | Anti-Rabbit | 1: 10 000 |
| p-PTEN (Ser380) | 54 | Cell Signaling Technology | #9551 | Rabbit | 1: 1000 | Anti-Rabbit | 1: 10 000 |
| PI3K p110 | 110 | Cell Signaling Technology | #4254 | Rabbit | 1: 1000 | Anti-Rabbit | 1: 10 000 |
| PI3K p85 | 85 | Abcam | #ab86714 | Mouse | 1: 1000 | Anti-Mouse | 1: 10 000 |
| p-PI3Kp85 (Tyr458) | 85 | Elabscience | #ENP0224 | Rabbit | 1: 1000 | Anti-Rabbit | 1: 10 000 |
| Akt | 60 | Abcam | #ab32505 | Rabbit | 1: 1000 | Anti-Rabbit | 1: 10 000 |
| p-Akt (Ser473) | 60 | Cell Signaling Technology | #4060 | Rabbit | 1: 1000 | Anti-Rabbit | 1: 5 000 |
| p-Akt (Thr308) | 60 | Cell Signaling Technology | #9275 | Rabbit | 1: 1000 | Anti-Rabbit | 1: 5 000 |
| PDK1 | 58-68 | Cell Signaling Technology | #3062 | Rabbit | 1: 1000 | Anti-Rabbit | 1: 10 000 |
| p-PDK1 (Ser241) | 58-68 | Cell Signaling Technolgy | #3061 | Rabbit | 1: 1000 | Anti-Rabbit | 1: 10 000 |
| mTOR | 289 | Abcam | #ab51089 | Rabbit | 1: 1000 | Anti-Rabbit | 1: 5000 |
| p-mTOR (Ser2448) | 289 | Abcam | #ab84400 | Rabbit | 1: 1000 | Anti-Rabbit | 1: 5000 |
| cRaf | 74 | Cell Signaling Technology | #9422 | Rabbit | 1: 1000 | Anti-Rabbit | 1: 10 000 |
| p-cRaf (Ser259) | 74 | Cell Signaling Technology | #9421 | Rabbit | 1: 1000 | Anti-Rabbit | 1: 10 000 |
| ERK1/ERk2 | 42, 44 | Cell Signaling Technology | #4695 | Rabbit | 1: 1000 | Anti-Rabbit | 1: 10 000 |
| p-ERK1/ERK2 (Thr202/Tyr204) | 42/44 | Cell Signaling Technology | #4370 | Rabbit | 1: 1000 (WB)  1:200 (IHC) | Anti-Rabbit | 1: 5000 (WB)  1:100 (IHC) |
| p38 | 43 | Cell Signaling Technology | #9212 | Rabbit | 1: 1000 | Anti-Rabbit | 1: 10 000 |
| p-p38 (Thr180/Tyr182) | 43 | Cell Signaling Technology | #9211 | Rabbit | 1: 1000 | Anti-Rabbit | 1: 10 000 |
| JNK | 46, 54 | Cell Signaling Technology | #9252 | Rabbit | 1: 1000 | Anti-Rabbit | 1: 10 000 |
| p-JNK (Thr183/Tyr185) | 46, 54 | Cell Signaling Technology | #9251 | Rabbit | 1: 1000 | Anti-Rabbit | 1: 10 000 |
| Bcl-2 | 26 | Santa Cruise | #130308 | Mouse | 1: 200 | Anti-Mouse | 1: 5000 |
| Caspase 9 | 51 | Cell Signaling Technology | #9508 | Mouse | 1: 1000 | Anti-Mouse | 1: 10 000 |
| Caspase 8 | 57 | Abcam | #ab25901 | Rabbit | 1: 1000 | Anti-Rabbit | 1: 10 000 |
| c-Caspase 8 | 18 | Abcam | #ab25901 | Rabbit | 1: 1000 | Anti-Rabbit | 1: 10 000 |
| Caspase 3 | 35 | Cell Signaling Technology | #9665 | Rabbit | 1: 1000 | Anti-Rabbit | 1: 10 000 |
| c-Caspase 3 | 17, 19 | Cell Signaling Technology | #9664 | Rabbit | 1: 1000 | Anti-Rabbit | 1: 10 000 |
| c-Caspase 7 | 20 | Cell Signaling Technology | #8438 | Rabbit | 1:1000 | Anti-Rabbit | 1:10 000 |
| LC3-I/-II | 14, 16 | Cell Signaling Technology | #3868 | Rabbit | 1:1000 | Anti-Rabbit | 1:5000 |
| p62 | 62 | Abcam | #ab109012 | Rabbit | 1:1000 | Anti-Rabbit | 1:10 000 |
| p53 | 53 | Abcam | #ab26 | Mouse | 1:1000 | Anti-Mouse | 1:10 000 |
| p21 | 21 | Invitrogen | #AHZ0422 | Mouse | 1:250 (WB)  1:200 (IHC) | Anti-Mouse | 1:5000 (WB)  1:250 (IHC) |
| p16 | 16 | Abcam | #ab189034 | Rabbit | 1:1000 | Anti-Rabbit | 1:10 000 |
| MCM2 | 125 | Abcam | #ab108935 | Rabbit | 1:1000 | Anti-Rabbit | 1:10 000 |
| PDGFRα | 190 | Cell Signaling Technology | #3174 | Rabbit | 1:1000 | Anti-Rabbit | 1:10 000 |
| α-SMA | 42 | Abcam | #ab7817 | Mouse | 1:1000 | Anti-Mouse | 1:5000 |
| E-cadherin | 97 | Cell Signaling Technology | #3195 | Rabbit | 1:1000 | Anti-Rabbit | 1:10 000 |
| Snail | 26 | Cell Signaling Technology | #3879 | Rabbit | 1:1000 | Anti-Rabbit | 1:10 000 |
| Vimentin | 54 | Cell Signaling Technology | #5741 | Rabbit | 1:1000 | Anti-Rabbit | 1:10 000 |

**Table S2.** Characteristics of mice

| **Characteristics** | **TC (n=15)** | **LD-DXR (n=16)** | **HD-DXR (n=16)** | **Total (n=47)** |
| --- | --- | --- | --- | --- |
| Weight (g) | 21.853333 ±  0.329156 | 21.58125 ±  0.5262796 | 22.25625 ±  0.3643239 | 21,8978723 ±  0,24532735 |
| Sex | Female | | | |
| Genotype | Wildtype | | | |
| Status | Healthy | | | |
